# Atrial Late Activation Mapping Predicts the Critical Isthmus of Left Atrial Re-entrant Tachycardia

**DOI:** 10.19102/icrm.2023.14092

**Published:** 2023-09-15

**Authors:** Hikmet Yorgun, Cem Çöteli, Kudret Aytemir

**Affiliations:** ^1^Department of Cardiology, Faculty of Medicine, Hacettepe University, Ankara, Turkey; ^2^Department of Cardiology, Cardiovascular Research Institute Maastricht (CARIM), Maastricht University Medical Center+, Maastricht, The Netherlands

**Keywords:** Atrial tachycardia, isochronal late activation mapping, re-entry

## Abstract

Functional atrial mapping is an emerging mapping modality to predict potential critical sites with a role in the maintenance of tachycardia. We report a case of atrial late activation mapping under sinus rhythm predicting the critical isthmus of a left atrial tachycardia. Our findings demonstrate the utility of an atrial isochronal late-activation mapping approach to predict the critical isthmus of re-entry.

Atrial tachycardia (AT) is a common rhythm disorder, especially in atrial fibrillation (AF) patients with low-voltage areas, attributed to either previous atrial substrate ablation or a de novo scar. High-density activation mapping is a useful method to understand the tachycardia mechanism; however, not all ATs are inducible during the electrophysiological study. Voltage mapping provides important data regarding the underlying atrial substrate to predict critical sites of tachycardia, but the functionality of such a substrate having a role in arrhythmia mechanisms has yet to be confirmed.

Here, we report a case of recurrent AT/AF in a 65-year-old female patient with previous cryoballoon ablation for persistent AF. Voltage mapping using 3-dimensional electroanatomic mapping (CARTO^®^; Biosense Webster, Diamond Bar, CA, USA) revealed low-voltage areas in the anterior wall and the roof of the left atrium with isolated pulmonary veins (PVs) **(Figure 1A)**. Isochronal late activation mapping (ILAM) during sinus rhythm using a multispline high-density catheter (PENTARAY^®^; Biosense Webster) demonstrated a deceleration zone (DZ) on the anterior wall with continuous fragmented signals in the latest activated zone **(Figure 1B)**. Briefly, ILAM was created during sinus rhythm manual annotation at the offset of the latest atrial deflections from the baseline. The total atrial activation window was set between the onset and the offset of the earliest local electrogram (EGM), which was divided into 8 equally distributed isochrones. DZs were defined as the areas with >3 isochronal colors within a 1-cm radius. Programmed stimulation induced a localized figure-of-8 re-entry on the anterior wall with fragmented EGMs at the critical isthmus, which was directly correlated with the DZ of ILAM **(Figure 1C)**. During mapping, episodic degeneration of AT into AF was observed. Ablation from this site terminated AT. An anterior mitral line from the mitral annulus to the right superior PV was created, including the DZ of ILAM as well as fragmented EGMs in the low-voltage region **(Figure 1D)**. The patient was free of any AT/AF during the 6-month follow-up period.

The utility of functional substrate mapping for ventricular tachycardia has long been known to predict the critical isthmus of re-entry.^1^ In addition, recent reports have demonstrated the usefulness of atrial late activation mapping during sinus/paced rhythm to reveal the critical isthmus of ATs.^2,3^ Similar to these data, our findings corroborated the utility of an atrial ILAM approach to predict the critical isthmus of a re-entry circuit. Functional evaluation of the underlying substrate might be considered to tailor the ablation strategy in re-do procedures with isolated PVs.

## Figures and Tables

**Figure 1:**
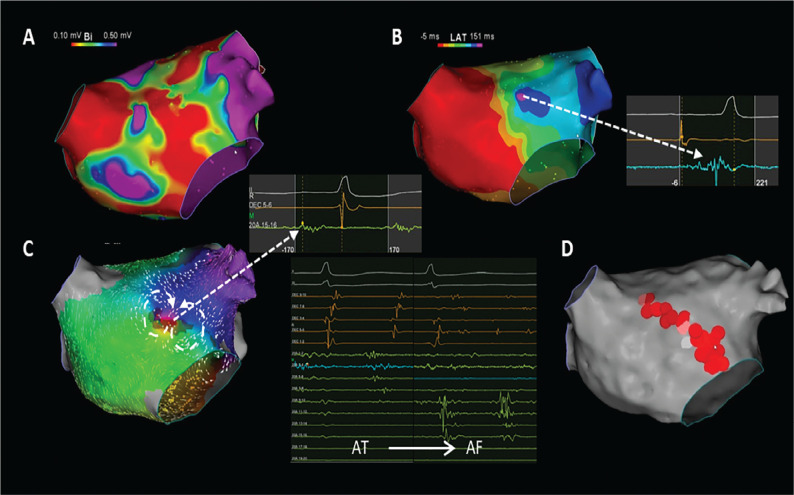
**A:** A left atrial voltage map demonstrating scar in the anterior wall with previous cryoballoon ablation history. **B:** Isochronal late activation mapping revealed deceleration zones with continuous fragmented morphology. **C:** The activation map demonstrated localized re-entry on the anterior wall, with continuous fragmented signals at the same site with deceleration zones during isochronal late activation mapping. Degeneration into atrial fibrillation also demonstrated continuous activity in the same site. **D:** A complete mitral line was created due to the presence of extensive fragmented electrograms throughout the anterior wall.

## References

[1] Aziz Z, Shatz D, Raiman M (2019). Targeted ablation of ventricular tachycardia guided by wavefront discontinuities during sinus rhythm: a new functional substrate mapping strategy. Circulation.

[2] Woods CE, Schricker AA, Nayak H (2022). Correlation between sinus rhythm deceleration zones and critical sites for localized reentrant atrial flutter: a retrospective multicenter analysis. Heart Rhythm O2.

[3] Kapur S (2021). Atypical flutter with atrial isochronal late-activation map correlating with the critical isthmus. J Innov Card Rhythm Manag.

